# Improvements in survival for patients with stage IV adenocarcinoma in the lung, diagnosed between 2010 – 2020 - A population-based registry study from Norway

**DOI:** 10.3389/fonc.2022.1017902

**Published:** 2022-11-29

**Authors:** Siri Børø, Steinar Thoresen, Åslaug Helland

**Affiliations:** ^1^ Institute of Clinical Medicine, University of Oslo, Oslo, Norway; ^2^ Merck (Norway), Oslo, Norway; ^3^ NordicRWE, Oslo, Norway; ^4^ Division of Cancer Medicine, Oslo University Hospital, Oslo, Norway

**Keywords:** stage 4 lung cancer, adenocarcinoma lung, epidemiology - descriptive, real world data (RWD), immunotherapy, survival %

## Abstract

**Objectives:**

We investigated how the prognosis for Norwegian patients with stage IV, adenocarcinoma (NSCLC) has developed during the last decade, to observe if increased survival coincides with the introduction of immunotherapy at a population level.

**Materials and methods:**

Incidence data from the Cancer Registry of Norway are virtually complete and includes information about histological subtypes and biomarkers. The data was used to analyze median and relative survival for females and males diagnosed with stage IV NSCLC, divided by histological subgroups and age-groups.

**Results:**

During 2010 – 2020, 14472 patients were diagnosed with lung cancer in stage IV, in Norway. Among them 6351 patients (43%) were classified with adenocarcinoma. The median survival has increased for both sexes, but the largest increase is seen in females. From 2010 to 2020, median survival for females in the 0-69 group increased from 6.7 months to 12 months and from 3.7 months to 10 months for the 70+ age group. For the equivalent male age groups, we see an increase from 6.1 months to 7.7 months for the 0-69 group, and an increase from 3.8 months to 4.5 months for the 70+ group. When excluding patients with EGFR/ALK mutations from the survival analysis, the groups continue to display an increased survival from 2010 to 2020, although modest in the male 70+ group. The 1-year relative survival (RS) has increased for both sexes, from 32.4% to 51.2 in females and 25.4% to 44.5% in males. When EGFR/ALK positive patients were excluded from the analysis 1-year RS in females rose from 32.4% to 47.4% and for males from 25.4% to 41.8%.

**Conclusion:**

A real-world patient population of stage IV, NSCLC adenocarcinoma have had a clinically meaningful increase in both median and relative survival from 2010 – 2020. The steepest survival increase has taken place after 2016, the time point where immunotherapy was implemented as a treatment option for the stage IV, adenocarcinoma population not harboring targetable mutations (EGFR/ALK).

## 1 Introduction

Approximately 85% of all lung cancers (ICD-C33, C34) are categorized as non-small cell lung cancer (NSCLC). NSCLC is further divided into subgroups where adenocarcinoma and squamous cell carcinoma are the most prevalent histological subtypes, counting for approximately 40% and 20%, respectively. A proportion of adenocarcinomas is characterized by so called “driver mutations”, which have consequences for treatment allocation. Epidermal growth factor gene, *EGFR*-mutation is the most common driver mutation, found in approximately 15% of NSCLC, and 5% are diagnosed with an anaplastic lymphoma kinase, *ALK*-alteration (1), although incidence varies by ethnicity (eg. around 30% EGFR mutations in the Asian NSCLC population (2)). As science progresses, we see an increasing list of genes that harbor specific activating aberrations that are targetable for molecules interfering with their signaling pathways.

Treating lung cancer patients against their targetable mutations in first-line (1L) has shown to benefit patients in terms of survival and increased quality of life and remains the standard of care according to international guidelines (3). Nevertheless, most patients with lung adenocarcinoma will *not* be present with driver mutations and requires another 1L treatment for their cancer. Since the year 2000 and up until 2016, platinum-based chemotherapy was the standard treatment for these patients in Norway (4). In 2016, immunotherapy, with Programmed Death- 1/Programmed Death ligand-1 (PD-1/PD-L1) inhibitors was first introduced for metastatic (stage IV) NSCLC patients with PD-L1 positive tumors in second-line (2L). PD-1/PD-L1 inhibitors have since been approved for reimbursement in Norway for all known subgroups of stage IV NSCLC, lacking driver mutations, in 1L setting.

While the survival for early-stage lung cancer has increased since the turn of the century, mainly explained by increase in those with localized stage and increased proportion of patients receiving curative intended treatment, survival of stage IV lung cancer has remained relatively poor in the same period (5). Nevertheless, with the introduction of immunotherapy the hypothesis is that a survival increase will be seen for stage IV NSCLC patients as well.

Immunotherapy has now been implemented in clinical practice for more than five years in Norway. The Cancer Registry of Norway (CRN) offers a unique opportunity to investigate survival on a population level as it contains incidence and survival information on all Norwegian cancer patients. Thus, we were able to investigate how survival of the stage IV NSCLC adenocarcinoma subgroup has developed during 2010 – 2020 in a complete real world (RW)-population. The aim was to observe if survival increased specifically after 2016, the time point of PD-1/PD-L1 inhibitors- introduction.

## 2 Materials and methods

### 2.1 The cancer registry of Norway

The Cancer Registry of Norway is a population-based registry established in 1951, and all health institutions involved in cancer care are required by law to report all cases of malignant neoplasm. The main sources of information to the CRN come from clinical notifications, pathology reports and death certificates, while the Norwegian Patient Registry (NPR) is an important additional source for identifying potentially unreported cancer cases. Information from the different sources is linked by the 11-digit personal identification number system that was established in Norway in 1964. Within the CRN lies clinical registries for specific cancers (including lung cancer) to provide a comprehensive overview of cancer specific diagnostics, treatment, and follow-up. Registration in the CRN is close to complete, and a comprehensive study in 2009 estimated the completeness to be 98.8% for the registration period 2001–2005 (6). The CRN assesses annually the degree of completeness and considers the data quality on the lung cancer patients to be particularly good, as they assess all pathology reports from all laboratories (7). As of 2020, the CRN contains information about 98,9% of all lung cancer patients in Norway (7). This includes histological types and biomarkers such as EGFR, ALK and PD-L1.

### 2.2 Diagnosis and staging

The Clinical Registry for lung cancer reports staging based on the TNM system (TNM version 8). Historically, the staging system reported by the CRN deviates from the TNM-system by not considering the tumor size. Thus, patients in TNM-stage I- III correspond to *localized* and *regional metastasis* respectively, while stage IV correspond to *distant metastasis*. Nevertheless, as of 2019 staging according to cTNM are published in the Annual lung cancer quality reports by the CRN (8). Tumor localization, classification of morphology and topography was coded according to ICD-10 and the International Classification of Diseases of Oncology (ICD-O).

### 2.3 Data collection and statistical methods

Data for this project was retrieved from the CRN database in October 2021. It included aggregated data from all patients diagnosed with stage IV lung cancer (C33 and C34) from 2010 to 2020 and the study periods were divided into calendar years. The data was used to analyze median and relative survival for females and males diagnosed with stage IV NSCLC, divided by histological subgroups and age-groups. The study focuses on the adenocarcinoma subgroup. To reduce the chance of random variation in survival analysis, the CRN has decided that the group size of each unit analyzed must include 30 or more patients. This study only used aggregated data and therefore not in scope for regional ethics approval.

For the analyses, the outcome was both median – and relative survival after being diagnosed with stage IV NSCLC, adenocarcinoma between 2010 - 2020. Follow-up was defined as the time from diagnosis to death or emigration, or up to November 4^th^ 2021 (date of data extraction). For females aged 0-69 diagnosed in 2020, an additional follow-up was done on June 28^th^, 2022, since they had not yet reached median survival upon the first date of data extraction. The incidence refers to the number of new cases. The method for estimating relative survival is based on the age-standardized Pohar-Perme method. For a detailed description of other statistical methods and requirements set out by the CRN, please see the Cancer in Norway-report, *Statistical methods* (9).

Patients present with EGFR- or ALK-mutations are excluded in the survival analysis of the group called *EGFRm+/ALKm+ excluded*.

The interpretation and reporting of these data are the sole responsibility of the authors, and no endorsement by the CRN is intended nor should be inferred.

## 3 Results

### 3.1 Demography, stage IV NSCLC, adenocarcinomas

#### 3.1.1 Number of cases, total population

According to the publicly available statistics bank within the CRN, between 2010 and 2020, 34 368 new cases of cancer in the lung (ICD-C33-34) were registered in Norway (10). The data shows that during that period, 14472 patients (ASR rate females: 22, ASR rate males: 28,9 (data not shown)), were diagnosed in stage IV which accounts for approximately 42% of all lung cancer cases diagnosed in Norway during the period. Among patients diagnosed in stage IV, 6351 patients (approximately 43%) had adenocarcinoma histology.

#### 3.1.2 Number of cases of stage IV patients with *EGFR* mutation and *ALK* translocation

*EGFR*- and *ALK* mutations are mainly found in patients with adenocarcinoma, and in Norway all patients with non-squamous NSCLC are routinely tested for these mutations. The number of stage IV, *EGFR*- and *ALK* positive (*EGFRm+/ALKm+*) patients increased from the beginning to the end of the period, see **Table 1**. However, *EGFR* mutation testing was implemented successively in Norway from June 2010. There is a possibility that the numbers from 2010 – 2015 is not reflecting the actual number of patients with positive *EGFR* status those years. The last four years of the period, *EGFR* mutation was registered for approximately 8 -10% of the adenocarcinoma patients, which is in line with the latest published quality reports by the CRN (7). Testing for *ALK* translocations was implemented in 2013, and the incidence rate seems to be relatively stable throughout the period, around 2-3%.

**Table 1:** Number of *EGFR* and *ALK positive stage IV* patients (*EGFRm+/ALKm+)*, 2010 – 2020.

	Number of cases of stage IV patients with *EGFRm+* or/and *ALKm+* pr year, sexes combined										
	2010	2011	2012	2013	2014	2015	2016	2017	2018	2019	2020
ALK	0 [0]	1[0.19]	0[0]	11[2.22]	15[2.71]	10[1.71]	12[2.19]	25[4.36]	12[2.08]	17[3.18]	15[2.62]
EGFR	0[0}	1[0.19]	11[2.11]	29[5.64]	36[6.26]	27[4.50]	47[8.07]	56[9.27]	64[10.16]	53[9.30]	60[9.71]

Proportion given in brackets [%].

### 3.2 Survival in patients with stage IV adenocarcinomas

#### 3.2.1 Median and relative survival, total population (*EGFRm+/ALKm+* included)

**Figure 1:**
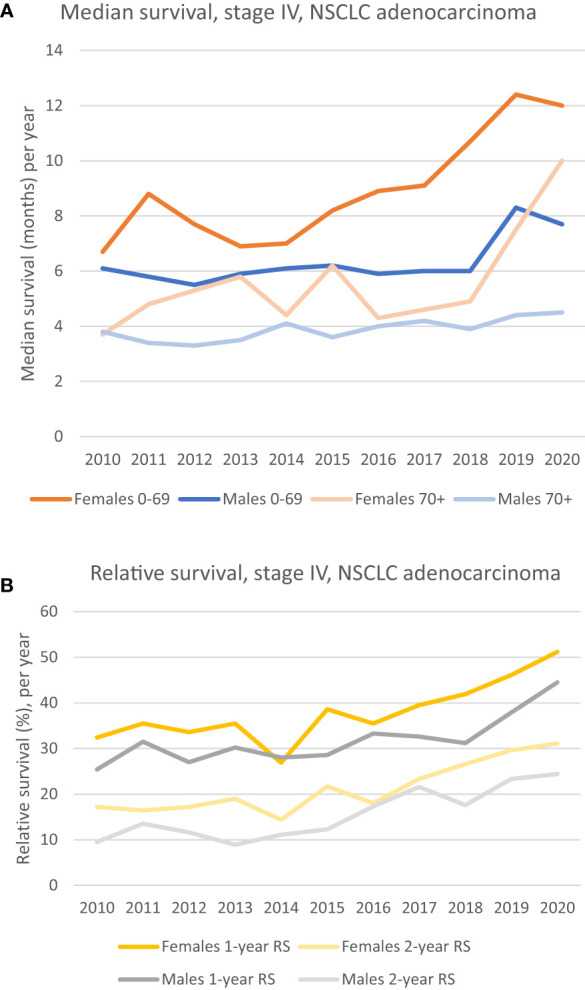
**(A)** Median survival of patients diagnosed in stage IV, NSCLC adenocarcinoma, by sex and age groups. Females aged 0-69 has had a steady increase since 2014, while the 70+ age group and the male 0-69 group show a marked increase in median survival from 2018. The median survival for males 70+ has been relatively poor and stable the whole period. **(B)** 1- and 2-year relative survival for patients diagnosed in stage IV, NSCLC adenocarcinoma. A clinically relevant improvement for both sexes can be seen. A unidirectional increase for females is seen from 2016, whilst it is seen from 2018 in males.

Taking the whole period (2010 – 2020) into consideration, the median survival of patients with stage IV adenocarcinoma has increased for both sexes in both age groups, 0-69 and 70+, see **Figure 1A**. Nevertheless, the median survival for males 70+ has remained stable and poor throughout the whole period, ranging from 3.8 months in 2010 to 4.5 months in 2020. For the male 0-69 group, fluctuations between 4 – and 6 months can be seen in the period of 2010 – 2018, but with a relatively steep increase from 2018 to 2019, jumping from respectively 6 months to 8.3 months (38.3% increase from one year to another). A clinically relevant increase in median survival for females is seen for both age groups. When considering the whole period, the 0-69 group had an increase of 79% (6.7 months to 12 months), and the 70+ group increased 170% (from 3.7 months to 10 months). Although the survival curve for the 0-69 female group gradually have increased from 2014, the steepest increase in survival seems to have occurred from 2017, while for the 70+ group, the steepest increase has taken place from 2018.

The relative survival (RS) of patients with stage IV adenocarcinoma has increased for both sexes, see **Figure 1B**. In 2020, 1-year RS for females was 51.2% vs 32.4% in 2010. For males the equivalent numbers are 44.5% in 2020 vs 25.4% in 2010. The 2-year RS for both females and males have the same positive trends throughout the period, with an increase from 17.2% in 2010 to 31.1% in 2020 for females, and an equivalent increase from 9.5% to 24.4% for males, see **Figure 1B**.

#### 3.2.2 Median survival, *EGFRm+/ALKm+ patients* excluded

**Figure 2:**
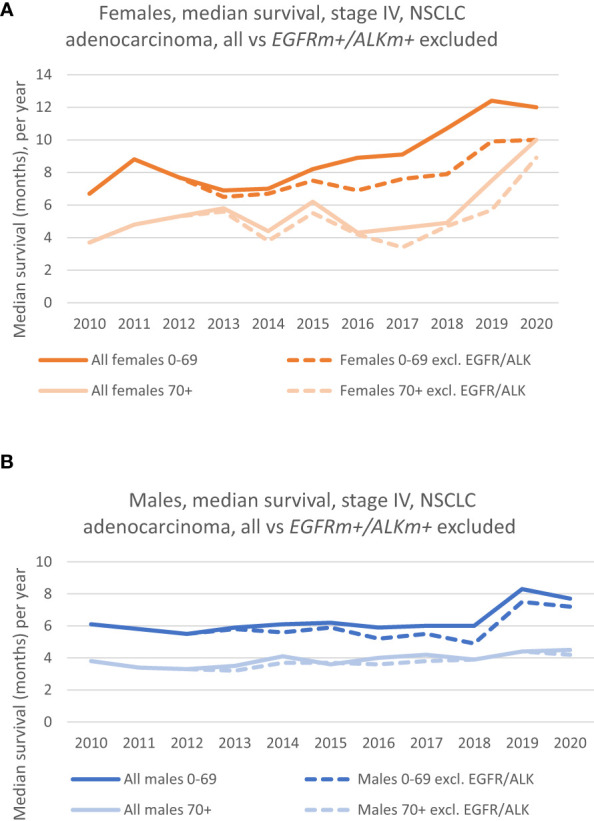
**(A)** Median survival of females, by age groups, where patients with *EGFRm*+/*ALKm+* (dotted lines) are excluded from the analysis. (Solid lines show the survival in the corresponding age group, where patients with driver mutations were included). **(B)** Median survival of males, by age groups, where patients with *EGFRm +/ALKm+* (dotted lines) are excluded from the analysis. (Solid lines show the survival in the corresponding age group, where patients with driver mutations were included).

The median survival for the patient group without EGFR/ALK mutations (*EGFRm+/ALKm+* excluded) shows the same positive trend throughout the period as for the whole patient group (*EGFR+/ALK+* included). The dotted lines in orange and light orange in **Figure 2A** shows how the median survival of females (*EGFRm+/ALKm+* excluded) have developed for the 0-69 group and 70+ group, respectively. It is evident that for females aged 0-69 the survival has increased during the decade of study, and the median survival in 2020 was 10 months compared to 6.7 months in 2010. A marked increase can especially be seen from 2018. For the 70+ age group (dotted lines) we also see the positive trend in increased survival. Median survival increased from 3.7 months in 2010 to 8.9 months in 2020.

The dotted lines (in dark blue and light blue) in **Figure 2B** shows the development in median survival for males (*EGFRm+/ALKm+* excluded), for the 0-69 group and the 70+ group, respectively. The group without patients harboring these mutations follow the same trend as the whole group (*EGFRm+/ALKm+* included), which is that any increase in median survival has been limited, especially for the 70+ group. Nevertheless, if we consider the whole period, there is an increase in median survival from respectively 3.8 months in 2010 to 4.2 months in 2020 for the 70+ group. For the 0-69 group, an increase in survival can particularly be seen from 2018, following the same trend as the *EGFRm+/ALKm+* included- group and had a median survival of 7.2 months in 2020 vs 6.1 months in 2010.

#### 3.2.3 Relative survival (*EGFR+/ALK+ patients* excluded)

**Figure 3:**
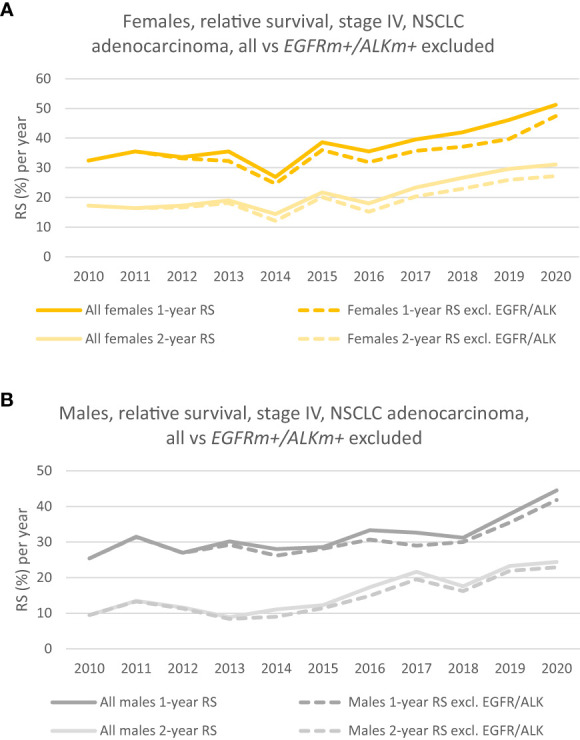
**(A)** 1- and 2-year relative survival of females, where patients with *EGFRm+/ALKm+* are excluded from the analysis (dotted lines). (Solid lines show the corresponding 1 -and 2-year RS for the group where patients with driver mutations were included). **(B)** 1- and 2-year relative survival of males where patients with *EGFRm+/ALKm+* are excluded from the analysis (dotted lines). (Solid lines show the corresponding 1 -and 2-year RS for the group where patients with driver mutations were included).

The relative survival (RS) has increased throughout the period for patients without *EGFR/ALK* mutations (*EGFRm+/ALKm+* excluded*)*. **Figure 3A** (dotted lines in yellow and light yellow) shows how respectively, the 1-year and 2-year RS of females without *EGFR/ALK* mutations has developed during the period. There is a steady increase in RS during the period, especially seen from 2016. The 1-year RS in 2020 was 47.4% vs 32.4% in 2010, whereas the 2-year RS follows the same positive trend and rose to 27.2% in 2020, from 17.2% in 2010. Dotted lines (in dark grey and light grey) in **Figure 3B** shows, respectively the 1-year and 2-year RS, for males without EGFR/ALK mutations. Even though the prognosis is poorer for males in all the measurements we’ve done, the trend in survival is net positive and the 1-year RS increased from 25.4% in 2010 to 41.8% in 2020. 2-year RS increased from 2010 to 2020 from respectively 9.5% to 22.9%.

## 4 Discussion

Evaluating RW-patient outcomes can both confirm or complement existing clinical trial data and fill potential knowledge gaps about patient performance in daily, clinical practice. Several PD-1 and PD-L1 inhibitors are now approved for the treatment of stage IV NSCLC. The results from especially the KEYNOTE-189-trial (KN-189) and KEYNOTE-024, (KN-024), demonstrated that introduction of immunotherapy as a 1L-therapy could have a positive effect on long term survival for non-squamous NSCLC patients without sensitizing EGFR-mutations or ALK-translocations (11, 12). Those respective studies demonstrated improved outcomes for patients receiving either immunotherapy as monotherapy or in combination with platinum-based chemotherapy + pemetrexed, compared to chemotherapy alone (11). But can we observe signs of improved survival on a population level after the national introduction of these agents? Norway has a publicly financed healthcare system, and all citizens are entitled to equal healthcare. Whenever a treatment for the Specialty care is assessed as cost-effective, the treatment will be implemented and made available to all (13), which should serve as a good basis to observe effects related to this, in the population.

The present population-based study found that the survival for stage IV, lung adenocarcinoma has increased during 2010 - 2020. The steepest increase in median survival has taken place between the years 2016 to 2020. This period coincides with the successive implementation of immunotherapy on a national scale, in Norway. The increase has been most substantial for females, but males in the age group 0-69 has also had an increase in median survival during the period. In line with several other studies showing that female, NSCLC patients demonstrated a decreased risk of progression and death, compared to males, so does our findings (14). Nevertheless, the aim of this study was to see *if*, and *when* a survival increase started to manifest in a population.

Howlader et al. argued that although they concluded that cancer treatment with immunotherapy either as monotherapy or in combination with chemotherapy undoubtedly had contributed to the decrease in population-level mortality, and substantially improved survival from NSCLC in the United States (US), the decline in mortality accelerated before immunotherapy was widely used, but occurred after routine testing for *EGFR* and *ALK* mutations was recommended in 2013, and the corresponding TKI (tyrosine kinase inhibitors)-targeted therapy was introduced in the US (15). This insinuates that both TKI-therapy and immunotherapy can contribute to increased survival in a population. We can draw similar conclusions from our current study as well. Even though the *EGFRm+* – or *ALKm+* patients constituted a small proportion of the stage IV, adenocarcinoma population in our study, respectively 8-10% and 2-3%, both median – and relative survival were consistently higher in the analysis where patients with *EGFRm+/ALKm+* were included. Although we have not evaluated treatment data, both EGFR-TKI inhibitors erlotinib and gefitinib were implemented in the Norwegian market when EGFR testing started in Norway in 2010 (16).

The CRN contains results from the EGFR-analyses mainly back to 2013. A study from 2012 reported the experiences with EGFR-testing in the first years after the implementation in 2010, that among 1058 NSCLC patients, 123 patients (11,6%) were *EGFR*-positive (16). For that study, the researchers had collected information directly from selected pathology laboratories from university hospitals. In **Figures 2A, B** and **3A, B** we see that the survival curves for respectively the *EGFRm+/ALKm+* - included and excluded, practically are overlaying each other from 2010 to 2012/2013. It might be that patients with *EGFRm+* in our dataset have not been identified during 2010 – 2013 (due to sparse CRN data quality), and consequently have not been properly excluded when we presented survival in the whole group vs survival in the group where *EGFRm+/ALKm+* patients were excluded. Nevertheless, this was accepted by the authors. If anything, the magnitude in survival difference from 2010 to 2020 for the stage IV, adenocarcinoma patients not harboring driver mutations is underestimated in our dataset.

More recently, according to the Annual quality reports for lung cancer in Norway, testing rate for EGFR mutations was 74.8% in 2017, 84.6% in 2018, 84.2% in 2019, and 84.4% in 2020 (7, 8, 17, 18). Although the prevalence of *EGFR*-mutations is reported to vary in different studies and populations, we expect the proportion of unknown *EGFRm+* patients to be low, as the proportions given in **Table 1** is in line with the proportion of *EGFRm+* NSCLC patients in comparable, European populations (19, 20).

This was a descriptive study, without the opportunity to do any causal relationship evaluation between treatment and outcome. However, one of the strengths of this study is that it is population based, and the analyses are based on our national registry. This enables the observation that an increase in survival occurred after a national implementation of immunotherapy, also when we removed the *EGFRm+/ALKm+* -patients from the analyses. Between 2010 to 2020, the only major change for the patient group without targetable mutations was treatment with PD-1/PD-L1 inhibitors in first – or later lines, thus it is plausible that the increased survival seen in the population could be an effect of the implementation of these treatments, in addition to biological differences between the sexes. Interestingly, when looking at the sub-group analysis of the KN-189 trial introduced earlier, it showed that the hazard ratio for death for females were remarkably 0.29 vs 0.70 for males, when patients were treated with the immunotherapy combination vs only chemotherapy (11). Further investigations should be done to understand potential sex-differences in survival of this patient group, as our real-world data also confirms that females have a higher survival rate than males.

The population in our study was unselected in the sense that it consisted of all stage IV, NSCLC adenocarcinoma patients throughout 2010 – 2020. Thus, any observed increase in survival cannot be attributed to patients being diagnosed at an earlier stage (down-staging) or being cured due to surgical techniques. This was on the other hand, the conclusion of another Norwegian study from 2016 who reported those reasons as cause for the observed increase in 5-year survival of Norwegian lung cancer patients between 2010 – 2016 (5). The same study concluded that survival outcome was practically unaltered and poor for patients with metastatic disease (5). Although an additional 3.1-, 4.7- and 2 months of increased survival between 2016 – 2020 for respectively, females aged 0-69, age 70+ and males aged 0-69, might seem modest, this increase occurred in a patient population with a poor prognosis.

Our study has some limitations. We are not able to exclude if the observed increase in survival is a result of a more “aggressive” treatment culture towards elderly patients in the latter years, compared to the years around 2010. Rather than older and maybe frail patients, most data from pivotal RCTs with immunotherapy comes from younger and fitter patients than those seen in the “real-world” (21). As a note, dividing the patients into the current age groups might have caused findings related to age to be more structural than of any biological importance, as several other studies have showed that patients younger than 65 years shows a better median survival value, than older patients (14). On the contrary, although there are no published clinical trials investigating the use of immune checkpoint inhibitors exclusively on elderly patients, a subgroup analysis of the Checkmate 017 study showed that patients aged 65–74 years had an almost similar improvement in the survival rate to that in patients younger than 65 years. Meanwhile, they did not observe any efficacy in patients 75 years or older, but point out that the treatment efficacy cannot be concluded to be inferior based on the analysis results, due to small sample size of elderly patients (22).

We cannot neglect the fact that implementation of PD-1/PD-L1-inhibitors in Norway has happened successively since 2016, leading to an ever-increasing patient population who potentially are eligible for PD-1/PD-L1 treatment throughout the period we studied. It started with the national reimbursement of pembrolizumab for stage IV, PD-L1 positive NSCLC patients in 2L in September 2016 (23), whereas the most recent immune checkpoint inhibitor reimbursement approval for NSCLC came in October 2021, with pembrolizumab for stage IV, 1L for squamous carcinoma NSCLC patients with low PD-L1 expression (24). Both Nivolumab and Atezolizumab have also been approved and implemented successively for subgroups in 2L- and 1L treatment, respectively. (Please see the **Supplementary Materials** for the detailed overview of the reimbursement of immunotherapy for NSCLC, in Norway, from 2016 until August 2021). This must be considered when interpreting the survival results and might explain why sub-groups display increased survival at different times and magnitude. This also means that patients diagnosed in the end of our period still has somewhat limited follow-up time, as long-term survival is defined as more than two years of survival after diagnosis (25).

At the time of data retrieval, the CRN was performing internal validation of their data on medicinal treatment, and it was not possible to further stratify the data presented, on different medical treatment regimes. We are thus cautious to attribute the observed, improved survival to immunotherapy, but our results shows that improved survival coincides in time with the national implementation of PD-1/PD-L1 inhibitors. From a report published by the CRN in 2021 we know that approximately 30% of NSCLC patients was treated with immunotherapy (mainly PD-1/PD-L1 inhibitors) in 2020, but also that approximately 25% of the patients did not receive any anti-cancer treatment at all (26). The opportunity to connect outcome with medical treatment, will add valuable real-world information on how patients respond to new drugs. This opportunity is now available at the CRN (11). Our study adds valuable insights on survival and demonstrates that the CRN can be used to further investigate the effect implementation of immunotherapy has had on a complete population in the real-world setting.

## 5 Conclusion

This study showed that a real-world patient population of stage IV, NSCLC adenocarcinoma have had a clinically meaningful increase in both median and relative survival from 2010 – 2020, and that the steepest survival increase has taken place after 2016. This was the time point when immunotherapy was implemented as a treatment option for the stage IV, adenocarcinoma population not harboring targetable mutations (*EGFR/ALK*). Further investigations should be done to understand the relatively large difference in survival between the sexes for this patient group and link medical treatment to the outcome measures.

## Data availability statement

The raw data supporting the conclusions of this article will be made available by the authors, without undue reservation.

## Ethics statement

Ethical review and approval was not required for the study on human participants in accordance with the local legislation and institutional requirements. Written informed consent for participation was not required for this study in accordance with the national legislation and the institutional requirements.

## Author contributions

SB: Conception and design of study. Acquisition of data, interpretation of data, manuscript writing. ST/ÅH: Advice and critically revision of manuscript for important intellectual content. All authors contributed to the article and approved the submitted version.

## Funding

This work was supported by the Norwegian Research Council [grant number 321291, 2021].

## Conflict of interest

SB is employed by Merck AB Norway. For 3 years since 2021 she holds an industrial PhD-position and during that time she is released from any mandatory work for Merck. The industrial PhD project is publicly financed by the Norwegian Research council, grant number 321291. She is enrolled as a PhD-student at the Medical Faculty at the University of Oslo, where her research focuses on the use of real-world data and how specifically Norwegian lung cancer registry data can be used as an external control arm for clinical trials. She is supervised by AH MD, Phd and ST MD, PhD. ST MD, PhD is the external supervisor of SB. He is an external consultant to Merck AB Norway and a founder of the company NordicRWE. This company has not been involved in the current study. AH MD, PhD is the internal supervisor of SB. In association with research/clinical studies she has received financial support and/or study drug from AstraZeneca, Roche, Novartis, Incyte, Eli Lilly, Ultimovacs and BMS. Adv board/advise: AstraZeneca, BMS, Janssen, MSD, Pfizer, Roche, Takeda, Sanofi, Bayer, EliLilly, Abbvie. All payments to institution. The funding bodies had no role in the data collection and analysis and were not involved in the interpretation of results, writing, revision, or approval of the manuscript.

## Publisher’s note

All claims expressed in this article are solely those of the authors and do not necessarily represent those of their affiliated organizations, or those of the publisher, the editors and the reviewers. Any product that may be evaluated in this article, or claim that may be made by its manufacturer, is not guaranteed or endorsed by the publisher.
