# Supplementary material for: Improvements in survival for patients with stage IV adenocarcinoma in the lung, diagnosed between 2010 – 2020 - A population-based registry study from Norway
Source: Front Oncol. 2022 Nov 29;12:1017902. doi: 10.3389/fonc.2022.1017902 (PMC9745181; doi:10.3389/fonc.2022.1017902)
Supplement: Supplementary file 1 [file Presentation_1.pdf]

Historical timeline of the drug approval and national reimbursement for PD-1/PD-L1 agents for stage IV NSCLC in Norway, from 2016 until Aug 30th 2021:

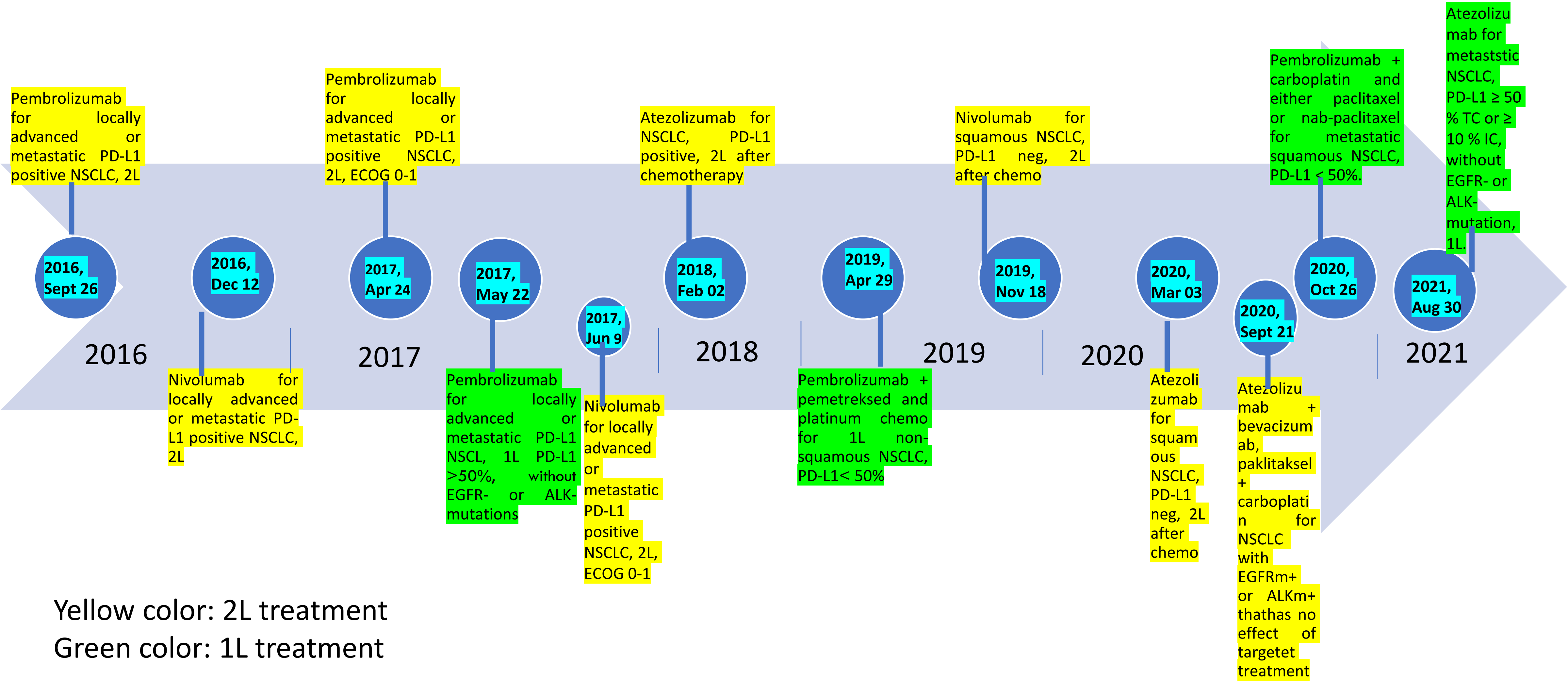

Yellow color: 2L treatment

Green color: 1L treatment
